# HPS6 Regulates the Biogenesis of Weibel–Palade Body in Endothelial Cells Through Trafficking v-ATPase to Its Limiting Membrane

**DOI:** 10.3389/fcell.2021.743124

**Published:** 2022-02-17

**Authors:** Jiran Lu, Jing Ma, Zhenhua Hao, Wei Li

**Affiliations:** Beijing Key Laboratory for Genetics of Birth Defects, MOE Key Laboratory of Major Diseases in Children, Rare Disease Center, National Center for Children’s Health, Beijing Children’s Hospital, Beijing Pediatric Research Institute, Capital Medical University, Beijing, China

**Keywords:** Weibel–Palade body, von Willebrand factor, lysosome-related organelle, Hermansky–Pudlak syndrome, HPS6, v-ATPase

## Abstract

The Weibel–Palade body (WPB) is one of the lysosome-related organelles (LROs) in endothelial cells, whose main content is von Willebrand factor (vWF). The biogenesis of LROs is regulated by the Hermansky–Pudlak syndrome (HPS) protein-associated complexes through transporting cargo proteins to WPBs. Our previous studies have shown that HPS6, a subunit of BLOC-2 complex, is likely involved in the maturation of WPBs. However, the underlying mechanism remains unknown. In this study, we found that the knockdown of *HPS6* in human umbilical vein endothelial cells (HUVECs) resulted in misshaped WPBs, decreased WPB number, and impaired vWF tubulation, which are similar to the characteristics of HPS6-deficient mouse endothelial cells. We observed similar morphological changes of WPBs in HUVECs after the knockdown of *ATP6V0D1* (a subunit of v-ATPase). Furthermore, we found that HPS6 interacted with ATP6V0D1, suggesting that HPS6 transports ATP6V0D1 to the WPB limiting membrane for the assembly of the v-ATPase complex to maintain its acidic luminal pH, which is critical for the formation of vWF tubules during WPB maturation. In conclusion, HPS6 likely regulates the biogenesis of WPBs by participating in the trafficking of v-ATPase to the WPB membrane.

## Introduction

Endothelial cells (ECs) form a layer of flat and polygonal cells that line the vascular wall, which is the biological barrier between the circulating blood and the blood vessel wall and is of great significance to maintain vascular homeostasis (Aird, 2007). The Weibel–Palade body (WPB) is a type of lysosome-related organelles (LROs) (Marks et al., 2013), which was discovered in 1964 by Ewald Weibel and George Palade in rat and human ECs (Weibel and Palade, 1964; Warhol and Sweet, 1984). As a morphological marker of ECs, WPBs are rod-shaped granules with a diameter of 0.1–0.3 μm and a length of 1–5 μm (Arribas and Cutler, 2000; Michaux and Cutler, 2004; Valentijn et al., 2008). WPBs contain various bioactive molecules, such as von Willebrand factor (vWF), angiopoietin-2, interleukin-6 and -8, monocyte chemoattractant protein-1, and P-selectin, which are released in response to the activation of ECs (Schillemans et al., 2019). ECs play an important role in numerous physiological activities that include hemostasis, inflammation, angiogenesis, and wound healing. The main luminal content of WPB is vWF. vWF forms a highly multimerized tubular structure that is closely related to the shape and size of WPBs (Wagner et al., 1982; Karampini et al., 2020). Upon release into the plasma, the vWF tubules will unfurl into long strings that recruit platelets to prevent bleeding (Savage et al., 2002).

As a member of LROs, WPB biogenesis is likely regulated by Hermansky–Pudlak syndrome (HPS) protein-associated complexes (HPACs), which includes the biogenesis of lysosome-related organelles complex (BLOC)-1, -2, and -3, adaptor protein complex-3 (AP-3), and homotypic fusion and protein sorting complex (Wei and Li, 2013). It has been shown that AP-1/clathrin coat plays an essential role in forming WPBs in endothelial cells (Lui-Roberts et al., 2005) and that the recruitment of CD63 is dependent on AP-3 complex during the biogenesis of WPBs (Harrison-Lavoie et al., 2006). However, the functions of other HPACs in WPB biogenesis are unknown yet.

In our previous study (Ma et al., 2016), we compared the phenotypes of WPBs and the secretion of vWF multimers in various HPAC mutant mice, including *pa* (HPS9 deficiency in BLOC-1), *ru* (HPS6 deficiency in BLOC-2), and *ep* (HPS1 deficiency in BLOC-3). We found that the regulated secretion of vWF in *ru* mice was the most significantly reduced, and the maturation of WPBs in *ru* mice was impaired significantly compared with that in wild-type (WT) mice. Therefore, we hypothesized that HPS6 may be required to deliver cargo proteins necessary for the biogenesis of WPBs.

In this study using *ru* ECs and human umbilical vein endothelial cells (HUVECs), we firstly confirmed the impairments of WPB biogenesis and maturation due to HPS6 deficiency. Considering that neutralization treatment does not alter the elongated shape of WPBs in *ru* ECs and vacuolar-type H^+^-ATPase (v-ATPase) is the primary proton pump for H^+^ homeostasis in organelles, we further found that HPS6 is required for the trafficking of subunit d1 in the V0 domain (ATP6V0D1) to WPBs to maintain the acidic luminal environment. Thus, HPS6 likely regulates the biogenesis of WPBs by transporting ATP6V0D1 to the WPB membrane to form highly ordered vWF tubules in the acidic lumen.

## Results

### Mutation of the *Hps6* Gene in Mice Impairs WPB Biogenesis and Maturation

**FIGURE 1:**
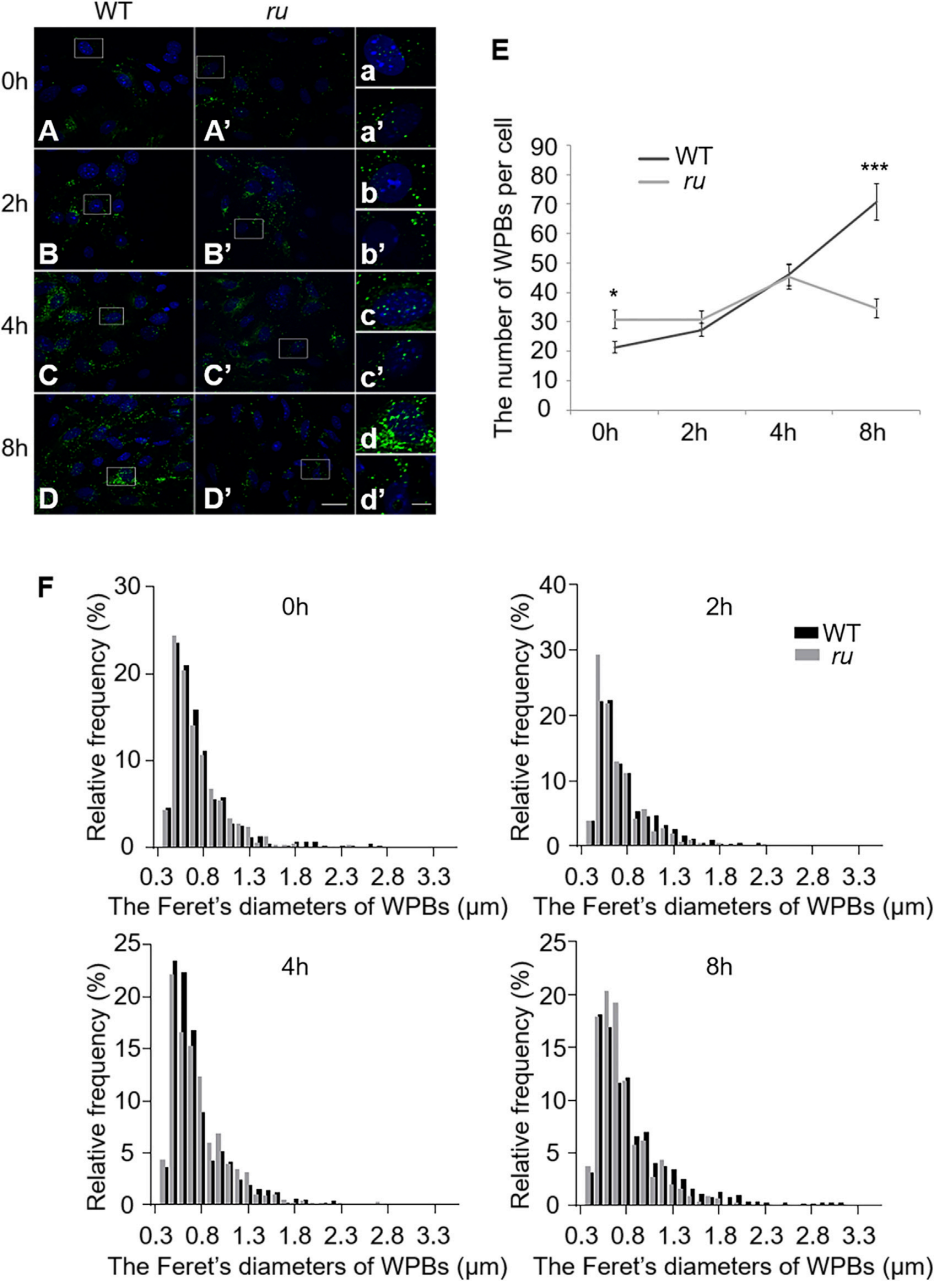
The number and the elongated shape of newly formed Weibel–Palade bodies (WPBs) are affected in *ru* endothelial cells (ECs). Primary endothelial cells were isolated from 4-week-old male WT and *ru* mice and cultured for 10 days, then 80 nM PMA was added to each dish to stimulate WPB secretion for 30 min, and the cells were fixed at 0, 2, 4, and 8 h after washing out. (**A**–**D**) (**A′**–**D′**) Immunofluorescence images of primary endothelial cells at different time points labeled against vWF (green) and nucleus (DAPI, blue). Scale bar, 20 μm. The boxed square in (**A**–**D**) and (**A′**–**D′**) was magnified as (a–d) and (a′-d′), respectively. Scale bar, 5 μm. (**E**) Quantitative analysis of the number of WPBs in each cell of WT and *ru* mice at different time points (*n* = 20, *p < 0.05, ***p < 0.001). (**F**) Feret’s diameter distribution of WPBs at each time point in WT and *ru* mice was analyzed quantitatively (WT_0h_: 428 WPBs, *ru*_0h_: 616 WPBs, WT_2h_: 545 WPBs, *ru*_2h_: 612 WPBs, WT_4h_: 921 WPBs, *ru*_4h_: 903 WPBs, WT_8h_: 1,412 WPBs, and *ru*_8h_: 692 WPBs). All the images were analyzed by the NIH ImageJ software. Data were expressed as mean ± SEM. Two independent experiments were performed.

Our previous study showed that WPBs in *ru*/*Hps6* mutant mice lose their classical “cigar” shape and do not release enough vWF multimers into the plasma after desmopressin stimulation (Ma et al., 2016). It is unknown whether these abnormities occur in the biogenesis stage or in the release stage. Therefore, we compared the shape and number of WPBs at various time points during their biogenesis between the primary ECs of WT and *ru* mice. The cells were treated with phorbol-12-myristate-13-acetate (PMA) to release existing mature WPBs. After washing out, the newly generated WPBs were examined at various time points (0, 2, 4, and 8 h). Immunofluorescence staining and statistical analysis showed that the average number of WPBs per cell in *ru* mice was higher than that in WT mice at 0-h time point after PMA treatment (Figures 1A,E) because there may be more immature WPBs that have not been released completely in *ru* mice compared with WT mice. When treated for a longer time, more WPBs that represented nascent WPBs were seen in WT cells than in *ru* cells. At 2 h, the number of WPBs in WT and *ru* ECs reached almost the same amount (*p* > 0.05). However, the number of WPBs in *ru* ECs was significantly reduced at 8-h time point compared with WT ECs (Figures 1B–E). The results of Feret’s diameter of WPBs in WT and *ru* mice showed a large cluster of WPBs in *ru* mice whose Feret’s diameter was concentrated at 0.5 μm and then reached the highest percentage (nearly 30%) at 2 h after PMA treatment. However, with the prolongation of time, WPBs in *ru* cells with Feret’s diameter of ≥1.3 μm were rarely seen and WPBs with Feret’s diameter of ≥1.8 μm were only seen in WT cells (Figure 1F), which indicated less mature WPBs in *ru* cells. Taken together, HPS6 deficiency may affect WPB biogenesis and maturation, which is consistent with our previous report (Ma et al., 2016).

### Biogenesis and Maturation of WPBs Are Compromised in *HPS6* Knockdown HUVECs

**FIGURE 2:**
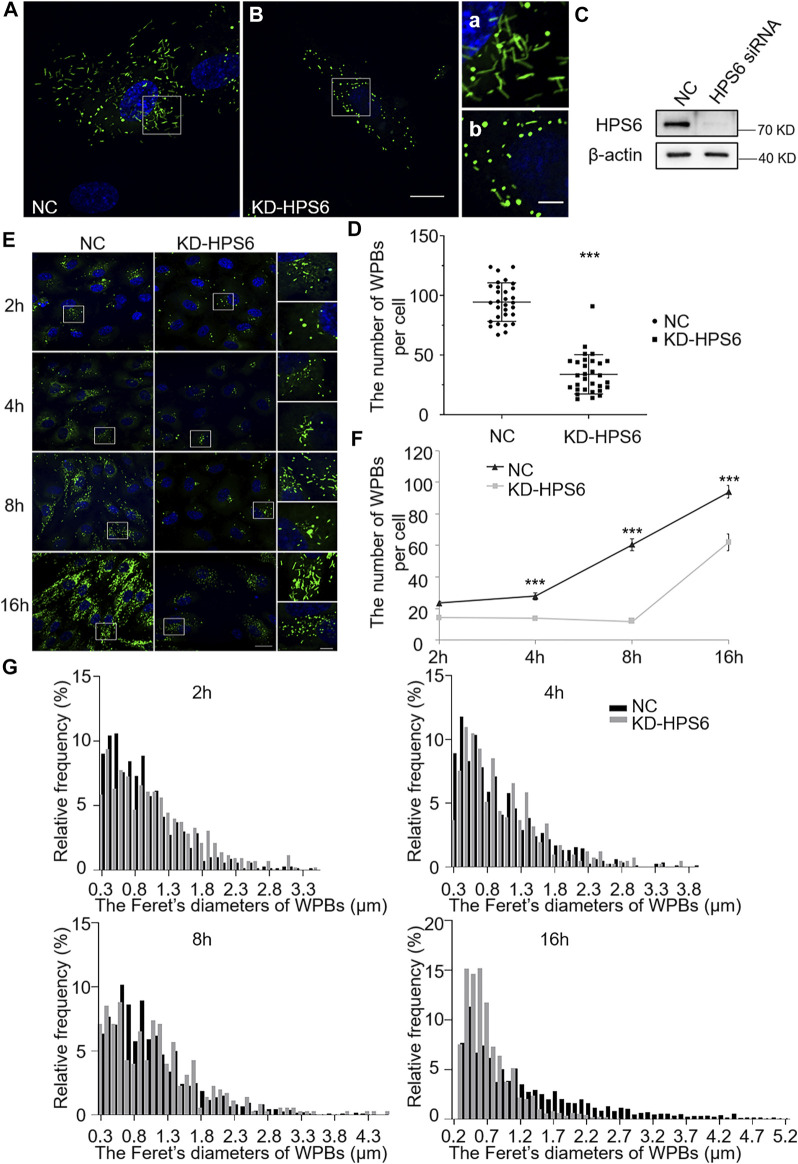
The number and the elongated shape of newly formed Weibel–Palade bodies (WPBs) are affected in KD-HPS6 human umbilical vein endothelial cells (HUVECs). (**A**, **B**) Immunofluorescence images of negative control (NC; **A**) and HPS6 (KD-HPS6; **B**) siRNA-mediated knockdown in HUVECs labeled against vWF (green) and nucleus (DAPI, blue). Moreover, 80 nM PMA was added to each dish to stimulate WPB secretion for 30 min, and the cells were fixed at 2, 4, 8, and 16 h after washing out. Scale bar, 20 μm. The boxed square in (**A**, **B**) was magnified as (a, b) respectively. Scale bar, 5 μm. (**C**) Western blotting analysis of the detection of *HPS6* knockdown. (**D**) Quantitative analysis of the number of WPBs per cell of NC and KD-HS6 HUVECs (*n* = 30, ***p < 0.001). (**E**) Both NC and KD-HPS6 HUVECs were exposed to 80 nM PMA for 30 min to stimulate WPB secretion, and the cells were fixed at 2, 4, 8, and 16 h, respectively, after washing out. Immunofluorescence images showed the HUVECs at different time points labeled against vWF (green) and nucleus (DAPI, blue). Scale bar, 20 μm. The boxed square was magnified, respectively. Scale bar, 5 μm. (**F**) Quantitative analysis of the number of WPBs per cell of NC and KD-HPS6 HUVECs (*n* = 20, ***p < 0.001). (**G**) Feret’s diameter distribution of WPBs at each time point in NC and KD-HPS6 HUVECs was analyzed quantitatively (NC_2h_: 700 WPBs, KD-HPS6_2h_: 427 WPBs, NC_4h_: 831 WPBs, KD-HPS6_4h_: 410 WPBs, NC_8h_: 812 WPBs, KD-HPS6_8h_: 352 WPBs, NC_16h_: 2,816 WPBs, and KD-HPS6_16h_: 1,862 WPBs). Data were expressed as mean ± SEM. Two independent experiments were performed.

We further investigated the effects of HPS6 deficiency by siRNA knockdown of *HPS6* (KD-HPS6) in HUVECs (Figure 2C). Immunofluorescence staining of vWF revealed more round-shaped WPBs in KD-HPS6 cells compared with negative control (NC) cells (Figures 2A,B). Moreover, the number of WPBs was significantly reduced in KD-HPS6 cells compared with NC cells (Figure 2D). These features resembled the changes in *ru* mouse ECs as we reported previously (Ma et al., 2016).

Similarly, we observed the newly formed WPBs in HUVECs at various time points (2, 4, 8, and 16 h) after PMA treatment (Figure 2E). Immunofluorescence staining and statistical analysis showed that the average number of WPBs per cell was significantly reduced in KD-HPS6 HUVECs at 4, 8, and 16 h after PMA treatment compared with the NC HUVECs (Figures 2E,F). In terms of the Feret’s diameter of WPBs in these two groups, the statistical results showed that the distribution of Feret’s diameter of WPBs in KD-HPS6 cells began to shift to small values from 4 h onward. At 16 h, there were significantly more WPBs with a Feret’s diameter of ≤1.5 μm in KD-HPS6 cells, while many rod-shaped WPBs of ≥2.5 μm were seen in NC cells (Figure 2G). These results confirmed that HPS6 deficiency affected the *de novo* production and maturation of WPBs.

### Regulated Secretion and Tubulation of vWF Are Compromised in *HPS6* Knockdown HUVECs

The existence of regular tubules of ultra-large vWF is an indicator of WPB maturation that is a driving force to form rod-shaped WPBs (McCormack et al., 2017; Tiemeier et al., 2020). Studies have shown that vWF is released by regulated secretion and unfurls into long strings that are highly efficient in recruiting platelets under flow in neutral plasma (Michaux et al., 2006; Ferraro et al., 2014; El-Mansi and Nightingale, 2021). However, string formation is also dependent on the initial coiling of vWF into tubules. Disruption of coiling impairs orderly unfurling and generates tangles of multimers, which fail to be released into the plasma. WPBs lose their classical rod shapes after the knockdown of *HPS6*. We speculated that this might result in failure of vWF multimerization.

**FIGURE 3:**
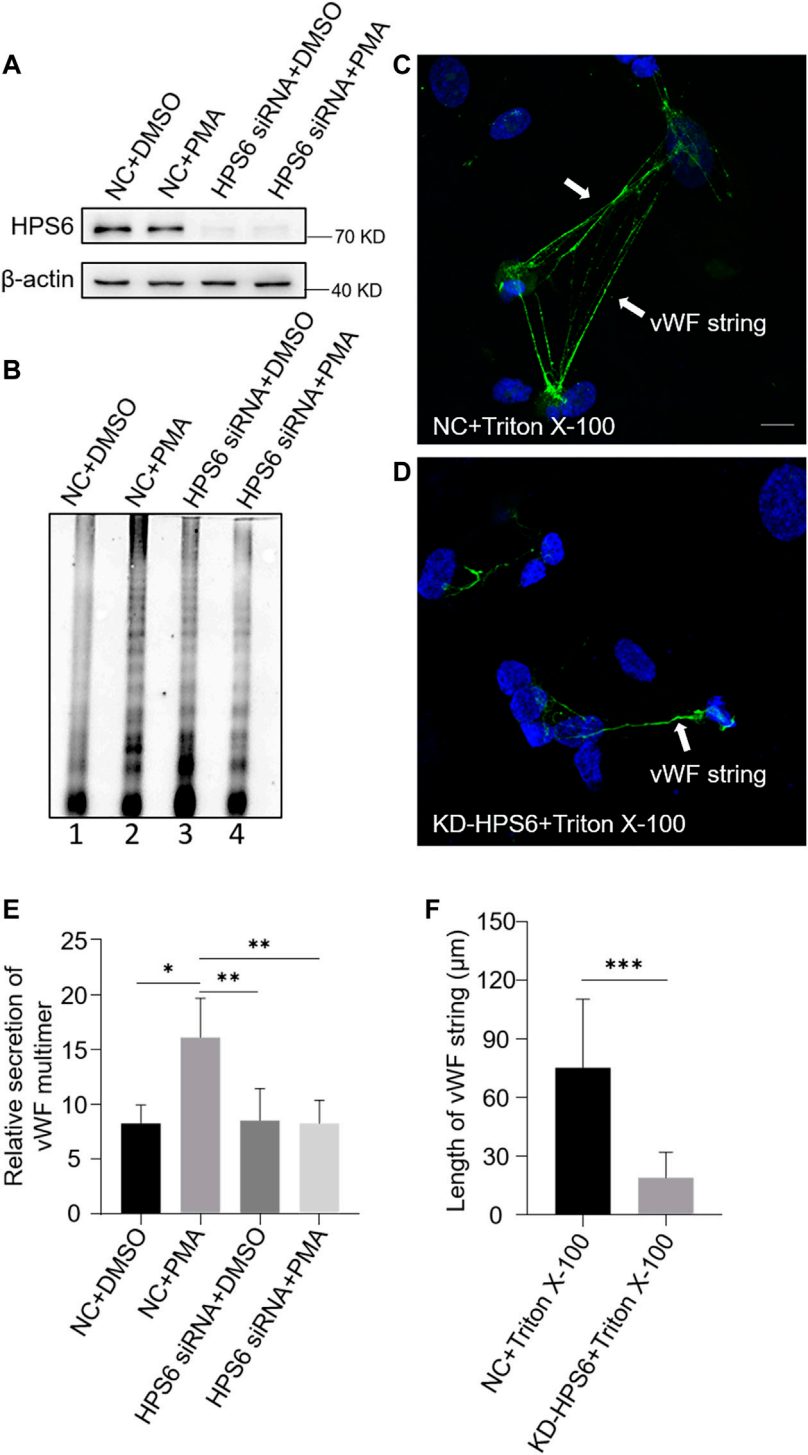
von Willebrand factor (vWF) secretion and the ability to generate surface strings are compromised in KD-HPS6 human umbilical vein endothelial cells (HUVECs). The negative control (NC) and HPS6 siRNA were transfected into two groups of HUVECs, respectively. At 72 h later, one group of NC and KD-HPS6 cells was exposed to 80 nM PMA for 30 min to stimulate WPB secretion, and the other groups were exposed to 0.1% DMSO instead. (**A**) Western blotting analysis of the detection of HPS6 knockdown in cell lysate collection. (**B**, **E**) Western blotting analysis of vWF multimer secretion in supernatant. The multimer gels were analyzed using the NIH ImageJ software. The quantification of supernatant vWF multimers was carried out based on the normalization of the β-actin protein level of the cells in each well. *n* = 5, *p < 0.05,**p < 0.01. (**C**, **D**) Moreover, 1% Triton X-100 was added into the culture medium of NC and KD-HPS6 HUVECs and cultured at 37°C for 1 h. Immunofluorescence images of two groups of HUVECs labeled against vWF (green) and nucleus (DAPI, blue) were shown. Scale bar, 10 μm. (**F**) The length of vWF strings at each group of HUVECs was measured by the NIH ImageJ software (*n* = 30 per group, ***p < 0.001). Data were expressed as mean ± SEM. Three independent experiments were performed.

We used PMA as a stimulus to compare the regulated secretion of vWF multimers between NC and KD-HPS6 HUVECs. The deficiency of HPS6 was confirmed by Western blotting analysis in KD-HPS6 HUVECs (Figure 3A). Western blotting with an anti-vWF antibody was used to analyze the culture supernatant that was subjected to SDS-agarose gel electrophoresis. The results showed apparently regulated secretion in NC cells after PMA stimulation, but not in KD-HPS6 cells (Figures 3B,E). These results suggested that HPS6 deficiency may lead to the failure of stimulated secretion of WPBs.

In general, VWF is secreted *via* three pathways. Regulated and basal secretion both occur from WPBs to transport highly multimerized VWF. Regulated secretion is triggered by agonist-mediated activation of the endothelium. In contrast, basal secretion occurs constitutively. The third pathway occurs by the constitutive release of VWF that has not been sorted into WPBs which is not subject to high multimerization. In Figure 3B, there was no difference between lane 1 (NC) and lane 3 (KD-HPS6), which can be explained by the fact that the constitutive release and the basal secretion of vWF did not seem affected after KD-HPS6. However, the comparison of lanes 1 and 2 *vs*. lanes 3 and 4 showed that the regulated secretion of vWF was compromised. Decreased vWF secretion is also one of the reasons why HPS6 deficiency leads to coagulation disorders.

To analyze the tubulation of vWF, the ability to form vWF strings in NC and KD-HPS6 HUVECs was assessed after treatment with Triton X-100. Triton X-100 destroys cell membranes and WPB membranes, which exposed vWF to neutral pH *in situ* (Michaux et al., 2006), thereby causing *in situ* diffusion to form vWF strings. Immunofluorescence staining showed vWF strings in NC cells, but very rarely in KD-HPS6 cells (Figures 3C,D). In addition, we measured the length of vWF strings in each group of HUVECs and found that the vWF strings were much shorter in KD-HPS6 cells than in WT cells (Figure 3F). This suggests that vWF multimers failed to unfurl into the plasma from KD-HPS6 HUVECs after stimulation, indicating failure of tubule formation in these cells. Thus, HPS6 plays an important role in the tubulation and secretion of vWF multimers in WPBs.

### Steady-State Levels of Several Subunits of v-ATPase Complex Are Decreased in HPS6 Knockdown Cells

**FIGURE 4:**
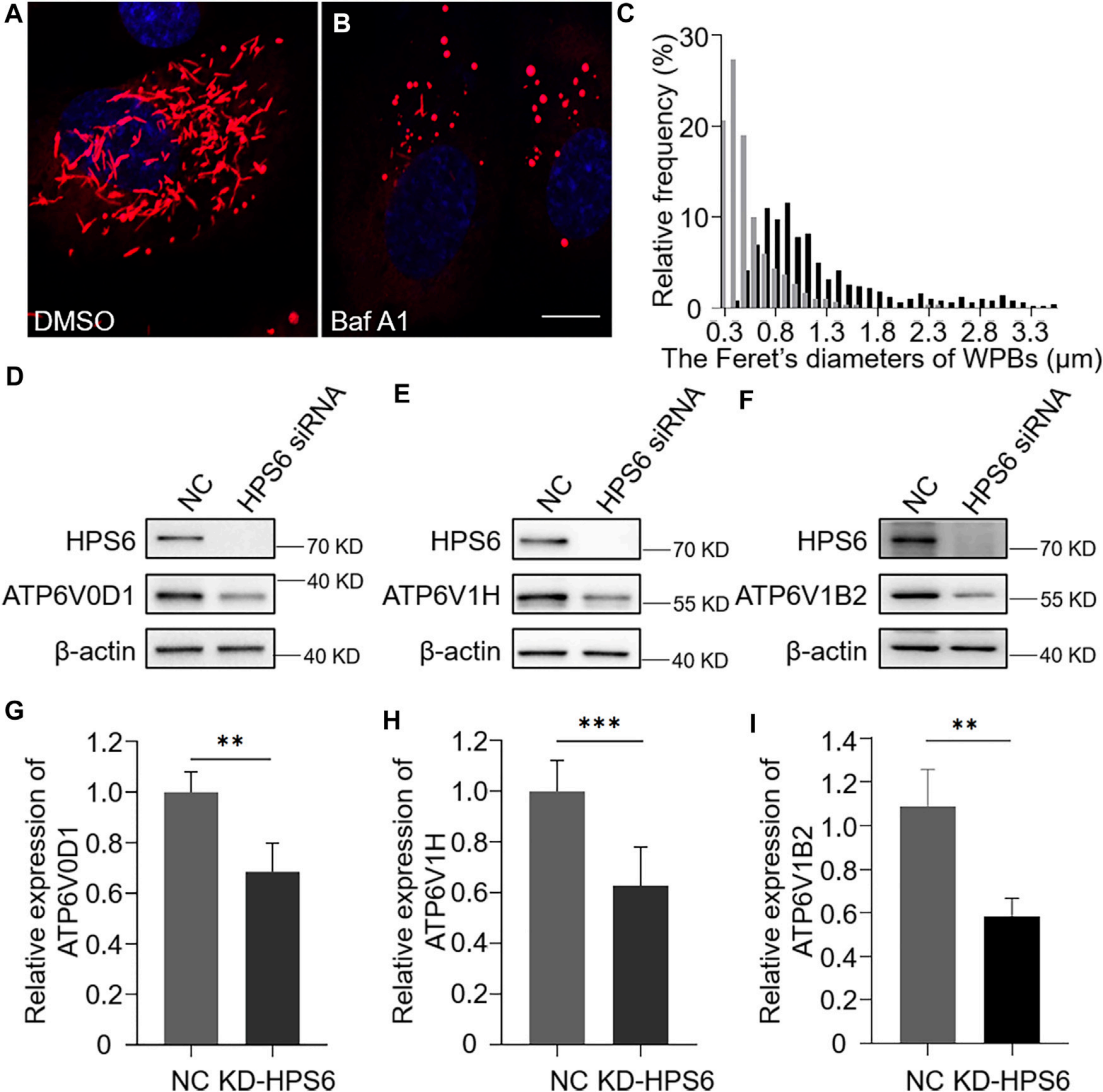
v-ATPase is compromised in KD-HPS6 human umbilical vein endothelial cells (HUVECs). Pharmacological inhibition of the v-ATPase caused the Weibel–Palade bodies (WPBs) to lose their elongated shapes. DMSO or v-ATPase inhibitor, Baf A1 (200 nM), was administered 1 h before cell fixation by 4% PFA (*n* = 20 per group). (**A**, **B**) Immunofluorescence images of DMSO-treated (**A**) and Baf A1-treated cells labeled against vWF (red) and nucleus (DAPI, blue). Scale bar, 10 μm. (**C**) Feret’s diameter of WPBs at each group of HUVECs was analyzed quantitatively (DMSO: 502 WPBs, gray; Baf A1: 300 WPBs, black). (**D**–**I**) siRNA-mediated HPS6 knockdown suppressed the expression of ATP6V0D1, ATP6V1H, and ATP6V1B2, three subunits of v-ATPase (*n* = 8 per group, **p < 0.01,***p < 0.001). Data were expressed as mean ± SEM. Three independent experiments were performed.

As vWF tubulation requires an appropriate acidic environment, we reason that the misshaped WPBs and abnormal release of WPBs in KD-HPS6 HUVECs might be due to a change of the pH value in the lumen of WPBs. It is well known that v-ATPase is the primary proton pump for H^+^ homeostasis in organelles, which consists of two multi-subunit domains, V1 and V0. The V1 domain consists of A, B, C, D, E, F, G, and H subunits, while the V0 domain consists of a, d, c, cʺ, e, and Ac45 subunits. It binds and hydrolyzes ATP for active intermembrane transport of protons (Futai et al., 2019). Therefore, we treated HUVECs with bafilomycin A1 (Baf A1), a v-ATPase inhibitor (Tang et al., 2019), to observe whether the WPB shape and Feret’s diameter changed accordingly. Our results showed that most WPBs in the Baf A1 group lost their elongated phenotype (Figure 4B), which was consistent with the phenotype of the KD-HPS6 group (Figure 2B), whereas no obvious abnormality was found in the DMSO control group (Figure 4A). The Feret’s diameter distribution of WPBs in these two groups showed that more WPBs in the Baf A1 group had a Feret’s diameter of ≤0.8 μm (Figure 4C), which indicated that v-ATPase inhibition significantly compromised the size of WPBs.

To further explore the effect of HPS6 on v-ATPase, the steady-state levels of several subunits of v-ATPase were examined in KD-HPS6 HUVECs by Western blotting. We found that the expression levels of ATP6V0D1, ATP6V1H, and ATP6V1B2 in the KD-HPS6 group were significantly lower than those in the NC group (Figures 4D–I). Similarly, these changes were observed in HPS3 (another BLOC-2 subunit) knockdown HUVECs (data not shown). These results suggest that HPS6 deficiency may destabilize the v-ATPase complex in KD-HPS6 HUVECs, which likely results in a higher pH in the lumen of WPBs and disrupts vWF tubule formation.

### HPS6 Interacts With ATP6V0D1 for Its Trafficking to WPBs

**FIGURE 5:**
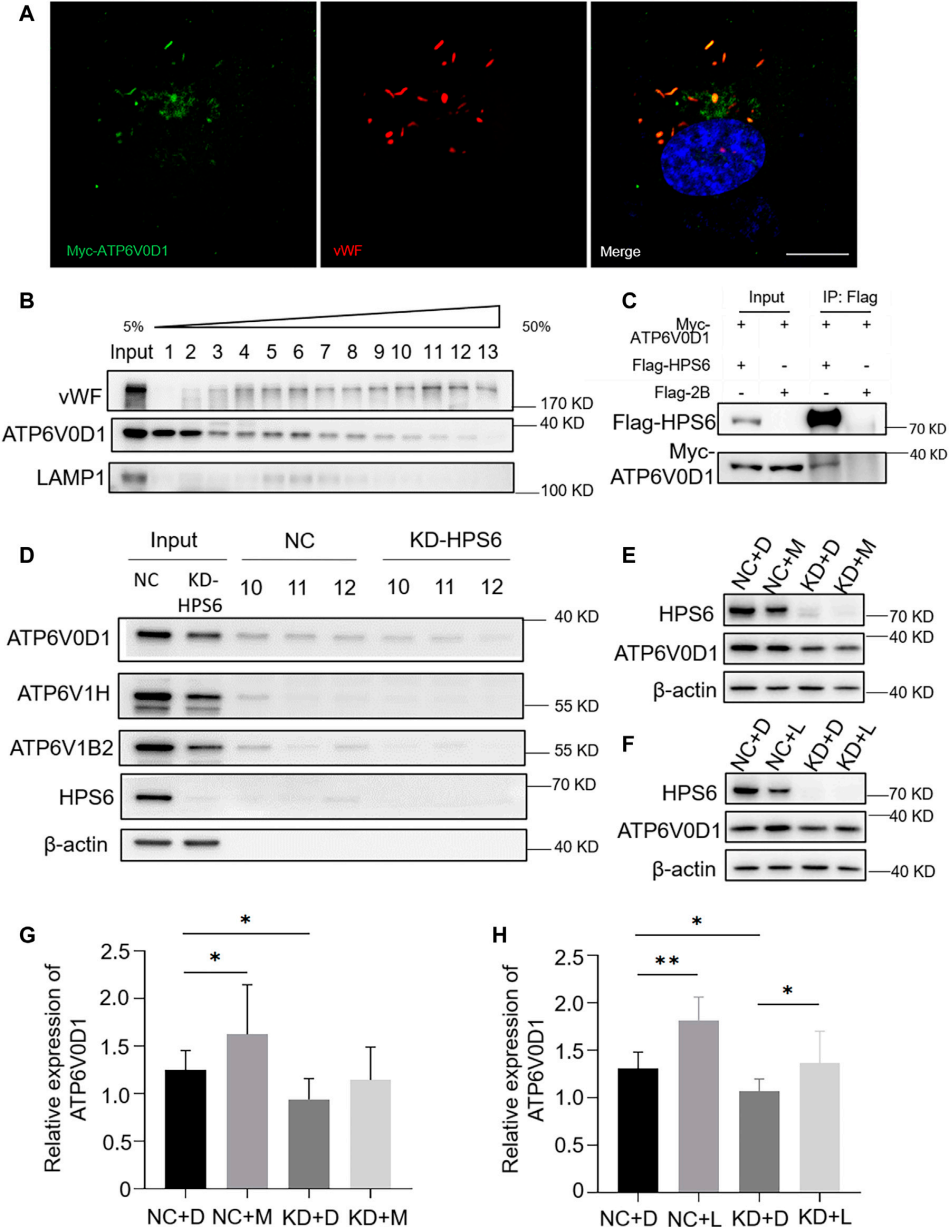
HPS6 interacts with ATP6V0D1 and mediates its trafficking to the Weibel–Palade body (WPB). (**A**) Immunofluorescence images of Myc-ATP6V0D1 plasmid-transfected human umbilical vein endothelial cells (HUVECs) labeled against Myc (green), vWF (red), and nucleus (DAPI, blue). Scale bar, 10 μm. (**B**) Separation of organelles and proteins by Optiprep density gradient. The final organelles and protein pellets were placed onto 50–5% sucrose gradient layers and then centrifuged for 30,000 rpm for 16 h at 4°C. A total of 13 fractions were collected from the top to the bottom of the gradient for further Western blotting experiments to test the distribution of vWF and ATP6V0D1. (**C**) A total of 293 T cells were transfected with Flag-HPS6 and Myc-ATP6V0D1 plasmids, and the cell lysates were co-immunoprecipitated by anti-Flag M2 affinity gel. Western blotting analysis of detection of Flag and Myc bands. (**D**) NC and HPS6 siRNA were transfected into two groups of HUVECs, respectively. At 72 h later, the organelles and proteins were separated by Optiprep density gradient. The input and fractions 10–12 of each group were analyzed by Western blotting. (**E**–**H**) The negative control (NC) and HPS6 siRNA were transfected into two groups of HUVECs, respectively. At 72 h later, one group of NC and KD-HPS6 cells was exposed to 0.25 μM MG132 (M) or 20 μM Leupeptin (L), and the other groups were exposed to 0.1% DMSO (**D**) instead. (**E**, **G**) Western blotting analysis of detection of ATP6V0D1 in KD + M cells. (**F**, **H**) Western blotting analysis of detection of ATP6V0D1 in KD + L cells. *n* = 7, *p < 0.05,**p < 0.01. Data were expressed as mean ± SEM. Two independent experiments were performed.

We next investigated whether v-ATPase subunits localized to WPBs. We observed a partial co-localization of the WPB marker vWF with Myc-tagged ATP6V0D1 (Figure 5A). To verify the subcellular localization, an Optiprep continuous density gradient was applied to examine the distribution of ATP6V0D1 and vWF in each fraction. The results showed that the main peak of vWF distribution was in the fractions 10–12, which may be the position of the mature WPBs, while the low-density fraction may represent the vWF in the ER, Golgi, and immature WPB. A proportion of ATP6V0D1 existed in the fractions 10–12, indicating that ATP6V0D1 is partially co-localized with mature WPBs. Part of ATP6V0D1 (fractions 5–8) was co-localized with late endosome/lysosome marker LAMP1 (Figure 5B). Furthermore, a co-immunoprecipitation analysis verified the interaction between Myc-tagged ATP6V0D1 and Flag-tagged HPS6 in 293 T cells (Figure 5C). Our endogenous immunoprecipitation (IP) also showed that HPS6 immunoprecipitated ATP6V0D1 (data not shown). These data suggest that ATP6V0D1 is likely transported to WPBs as a cargo of HPS6 or BLOC-2.

In order to compare the v-ATPase subunits on WPB after the knockdown of *HPS6*, we performed the Optiprep continuous density gradient experiment and took the same amount of fractions 10–12 that mainly represented the mature WPBs in NC and KD-HPS6 cells for Western blotting. The results showed that the protein levels of ATP6V0D1, ATP6V1H, and ATP6V1B2 were decreased in fractions 10–12 (Figure 5D). These results suggested that ATP6V0D1 could not be transported to the WPB membrane correctly after HPS6 deficiency, which likely affects the assembly of the v-ATPase complex and results in disrupted WPB acidification.

To explore whether the decrease in the protein level of ATP6V0D1 after *HPS6* knockdown is due to the degradation of the mis-localized protein, we treated NC and KD-HPS6 HUVECs with a proteasome inhibitor MG132 (M) (Mazzotta et al., 2020; Olmedo et al., 2020) and a lysosome inhibitor leupeptin (L) (Mazzotta et al., 2020), respectively. We optimized the concentration of 0.25 μM MG132 and 20 μM leupeptin as the experimental conditions. Our results showed that, although ATP6V0D1 was increased significantly in the NC + M and NC + L groups compared with that in the NC + D (DMSO) group (Figures 5E–H), a significant increase of ATP6V0D1 was found only in the KD + L group and not in the KD + M group compared with the KD + D group (Figures 5E–H). These results suggest that, although a portion of ATP6V0D1 may be degraded by the proteosome, ATP6V0D1 is mainly subjected to lysosomal degradation, indicating that mis-targeted ATP6V0D1 is likely degraded *via* the lysosomal pathway that results in a decrease of the steady-state level of ATP6V0D1.

### *ATP6V0D1* Knockdown Phenocopies the Abnormalities of WPBs in *ru* Mouse Cells and in KD-HPS6 HUVECs

**FIGURE 6:**
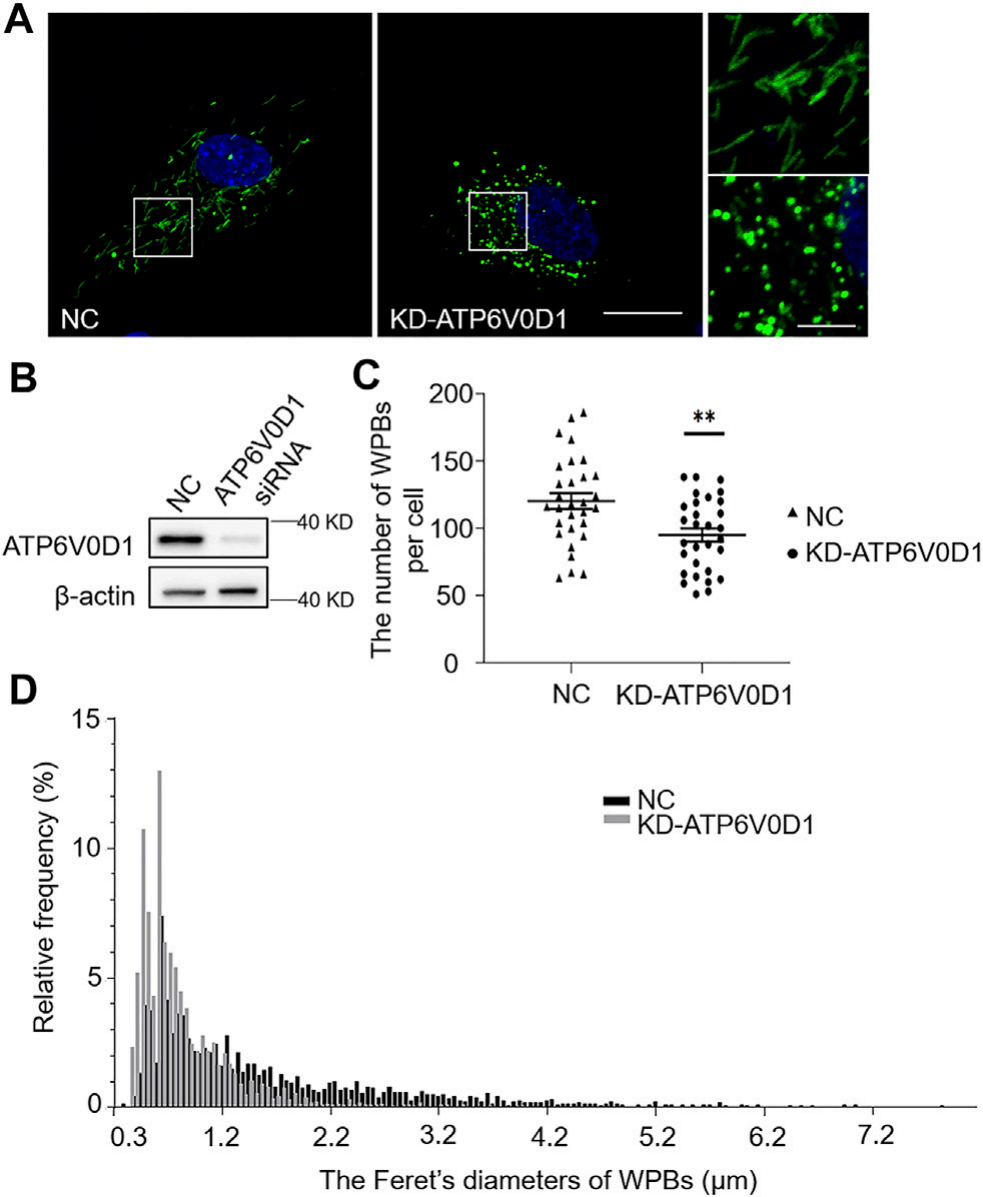
Knockdown of ATP6V0D1 in human umbilical vein endothelial cells (HUVECs) phenocopies the abnormalities of Weibel–Palade bodies (WPBs) KD-HPS6 HUVECs. (**A**) Immunofluorescence images of negative control (NC) and ATP6V0D1 siRNA (KD-ATP6V0D1)-mediated knockdown in HUVECs labeled against vWF (green) and nucleus (DAPI, blue). Scale bar, 20 μm. The boxed squares were magnified, respectively. Scale bar, 5 μm. (**B**) Western blotting analysis of the detection of ATP6V0D1 knockdown. (**C**) Quantitative analysis of the number of WPBs per cell of NC and KD-ATP6V0D1 HUVECs (*n* = 30, **p < 0.01). (**D**) Feret’s diameter distribution of WPBs at each group of HUVECs was analyzed quantitatively (NC: 3,608 WPBs, black; KD-ATP6V0D1: 2,852 WPBs, gray). Data were expressed as mean ± SEM. Three independent experiments were performed.

**FIGURE 7:**
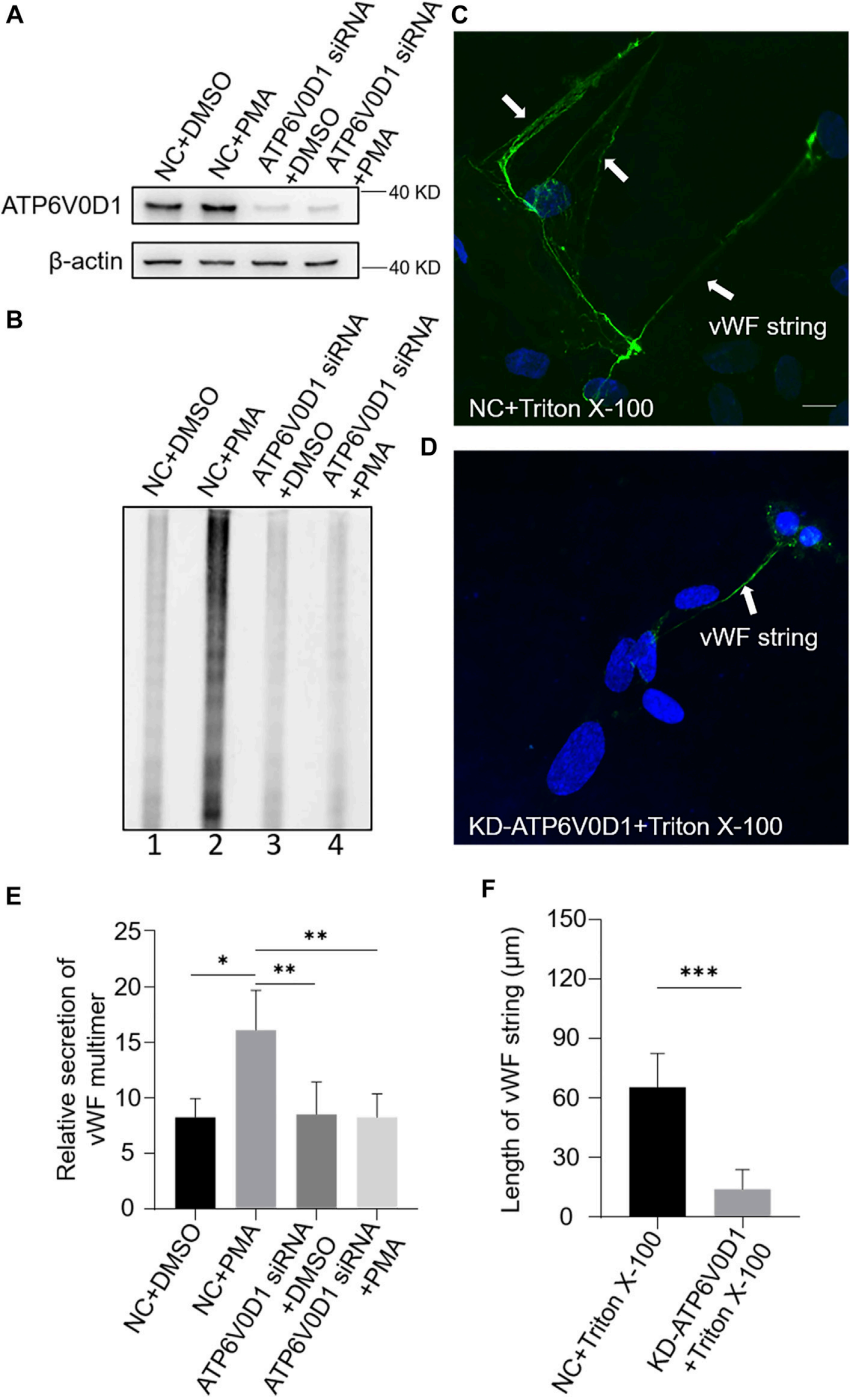
vWF secretion and the ability to generate surface strings are also compromised in KD-ATP6V0D1 human umbilical vein endothelial cells (HUVECs). Negative control (NC) and ATP6V0D1 siRNA were transfected into two groups of HUVECs, respectively. At 72 h later, one group of NC and KD-ATP6V0D1 cells was exposed to 80 nM PMA for 30 min to stimulate Weibel–Palade body secretion, and the other groups were exposed to DMSO (0.1%) instead. (**A**) Western blotting analysis of the detection of ATP6V0D1 knockdown in cell lysate collection. (**B**, **E**) Western blotting analysis of the detection of vWF multimer secretion in supernatant. Multimer gels were analyzed using the NIH ImageJ software. The quantification of supernatant vWF multimers was carried out based on the normalization of the β-actin protein level of the cells in each well. *n* = 5, *p < 0.05,**p < 0.01. (**C**, **D**) 1% Triton X-100 was added into the culture medium of NC and KD-ATP6V0D1 HUVECs and cultured at 37°C for 1 h. Immunofluorescence images of two groups of HUVECs labeled against vWF (green) and nucleus (DAPI, blue) were shown. Scale bar, 10 μm. (**F**) The length of vWF strings at each group of HUVECs was measured by the NIH ImageJ software (*n* = 30 per group, ***p < 0.001). Data were expressed as mean ± SEM. Three independent experiments were performed.

We next investigated the changes of WPBs in HUVECs after the knockdown of the *ATP6V0D1* gene (KD-ATP6V0D1). We found that the changes in shape, number, and size of WPBs in KD-ATP6V0D1 HUVECs were similar to those in *ru* mouse ECs and KD-HPS6 HUVECs (Figures 6A–D). The off-target effects of both *siHPS6* and *siATP6V0D1* were excluded by three different target siRNAs (data not shown). Additionally, the regulated secretion and ability to unfurl into the plasma of vWF multimers were impaired in KD-ATP6V0D1 HUVECs (Figure 7). These results further confirmed that HPS6 is likely involved in the trafficking of ATP6V0D1 to the WPB membrane for the assembly of v-ATPase to maintain the acidic pH in its lumen. This highlights the importance of H^+^ homeostasis in the lumen of WPBs during their biogenesis and release.

## Discussion

The BLOC-2 complex functions in transporting cargos from endosomes to lysosomes or lysosome-related organelles in cells. Many studies have shown that the BLOC-2 complex plays an important role in the biogenesis of melanosomes (Dennis et al., 2015). As one of the subunits of the BLOC-2 complex, HPS6 has been reported to be involved in lysosome positioning and maturation in HeLa cells (Li et al., 2014). In this study, we found that HPS6 is involved in the trafficking of ATP6V0D1, a subunit of v-ATPase complex, to the WPB membrane for its assembly into v-ATPase complex to maintain the acidic state of the WPB lumen, which is essential for vWF tubulation and the formation of elongated mature WPBs. When HPS6 is deficient, ATP6V0D1 is missorted and likely subjected to lysosomal degradation, which increases the pH in the lumen of WPBs and ultimately disrupts WPB biogenesis and secretion. This result explains one of the molecular mechanisms by which HPS6 participates in the biogenesis and maturation of WPBs.

Additionally, we revealed that the protein level of ATP6V1H and ATP6V1B2, two other subunits of v-ATPase, was significantly reduced in KD-HPS6 cells. v-ATPase is a macro-complex that consists of two domains with 14 subunits. Except for the ATP6V0D1 subunit, HPS6 or BLOC-2 complex may be similarly involved in the trafficking of other v-ATPase subunits to WPBs. Because of the lack of available antibodies, the interactions between HPS6 and other v-ATPase subunits require further investigation. Another explanation is that the lack of one subunit destabilizes the other subunits in a heterogeneous complex. This is evident in the HPS mutants of BLOC-1/-2/-3, *i*.*e*., one subunit deficiency destabilizes the other subunits (Wei and Li, 2013).

During WPB maturation, the luminal pH value is not constant, but it decreases gradually as the WPB matures. The direct correlation between this pH change and the shape of WPBs is evident by vWF multimerization and the assembly of the tubular structures. Mayadas *et al*. found that vWF dimers form multimers when the pH value is between 4.0 and 6.2, and the highest efficiency of multimerization occurs at 5.8 (Mayadas and Wagner, 1989). However, vWF multimers are no longer detectable when pH >6.2. Using compounds such as monensin, NH_4_Cl, and chloroquine to increase the pH in the medium, obvious phenotypic changes are visible under an electron microscope, and WPBs showed a significantly round shape (Michaux et al., 2006), which are consistent with the phenotypic changes of WPBs in KD-HPS6 HUVECs. This agrees with the notion that the luminal pH of WPBs is important for their maturation. Unfortunately, we have tested several dyes or tools to monitor the luminal pH changes but failed. A sensitive tool for this measurement needs to be developed.

It is known that the highly polymerized vWF polymers are stored in mature WPBs, which dock near the plasma membrane and release their contents into the blood after stimulation. Both in the endothelial cells of *ru* mice and HUVEC cells, HPS6 deficiency not only changed the classic long rod shape of WPB but also led to a significant reduction in the number of newly generated WPBs. One possible explanation is that HPS6 may also participate in the process of WPB budding from TGN, which awaits further investigation.

In summary, we have revealed that HPS6 or BLOC-2 is involved in the trafficking of v-ATPase subunits to maintain the acidic lumen of LROs. This provides more insights into the underlying mechanism of LRO defects and the pathogenesis of HPS.

## Materials and Methods

### Antibodies

Antibodies Used in Immunofluorescence

The mouse monoclonal antibodies anti-vWF antibodies (1:500, ab201336) were purchased from Abcam (United Kingdom). The rabbit polyclonal anti-human vWF antibodies (1:2,000, A0082) were purchased from Dako (Denmark). The rabbit polyclonal anti-c-Myc antibodies (1:500, C3956) were purchased from Sigma-Aldrich (United States). The goat anti-rabbit IgG (H + L) Highly Cross-Adsorbed Secondary Antibody, Alexa Fluor 488 (1:2,000, CA11008s) or Alexa Fluor 594 (1:2,000, CA11012s) and goat anti-mouse IgG (H + L) Highly Cross-Adsorbed Secondary Antibody, Alexa Fluor Plus 488 (1:2,000, CA11001s) were purchased from Invitrogen (United States).

Antibodies used in Western blotting

The rabbit polyclonal anti-human vWF antibodies (1:10,000, A0082) were purchased from Dako (Denmark). The rabbit polyclonal antibodies against human HPS6 (1:1,000, NBP2-14,100) were from Novus Biologicals (United States). The rabbit polyclonal antibodies against human ATP6V1B2 (1:500, ab73404), ATP6V1H (1:500, ab187706), and ATP6V0D1 (1:1,000, ab202899) were all from Abcam (United Kingdom). The mouse monoclonal antibodies anti-human β-actin (1:100,000, A5441), rabbit polyclonal anti-c-Myc antibodies (1:1,000, C3956), and rabbit polyclonal anti-LAMP1 antibodies (1:2,000, L1418) were purchased from Sigma-Aldrich (United States). The mouse monoclonal anti-Flag tag (1:1,000, MA1-91878) were purchased from Invitrogen (United States). The peroxidase-conjugated secondary anti-rabbit antibodies and anti-mouse antibodies (both 1:5,000, ZB2301 and ZB2305) were all purchased from ZSGB-bio (China).

### Animals

Male WT or ruby-eye (*ru*) mutant (HPS6 deficiency in BLOC-2) mice (4 weeks of age) were used for the experiments. They were originally obtained from The Jackson Laboratory (Maine, United States) and maintained in the laboratory of Dr. Richard T. Swank. All these mutant mice arose from spontaneous mutations in C57BL/6J background. The mutation of the *Hps6* gene was confirmed by PCR analysis of tail DNA (data not shown). The animal experiments were carried out in accordance with institutional guidelines for animal experimentation, and the procedures were approved by the Institutional Animal Care and Use Committee of the Institute of Genetics and Developmental Biology, Chinese Academy of Sciences (mouse protocol # AP2021028).

### Cell Culture

Primary mouse heart endothelial cells were isolated by magnetic activated cell sorting method as previously described (Ma et al., 2016). The human umbilical vein endothelial cells were obtained from Dr. Qiang Wang’s Laboratory (Institute of Zoology, Chinese Academy of Sciences) and maintained in EGM-2 medium (Lonza, cc-3162) at 37°C and 5% CO_2_.

### *ru* EC Treatments

Phorbol-12-myristate-13-acetate (Sigma, P1585) in DMSO was added into the EGM-2 medium with 80 nmol/L final concentration. The cells were washed with the growth medium twice after 30 min, changing to fresh growth medium, and then fixed with 4% paraformaldehyde at 0, 2, 4, and 8 h later, respectively.

### HUVEC Treatments

PMA in 80 nM final concentration or 0.1% DMSO was added into the EGM-2 medium (respectively) after 30 min. The cells were washed with growth medium twice, changing to fresh growth medium, and then fixed with 4% PFA after 2, 4, 8, and 16 h, respectively, or the supernatant was collected directly. Triton X-100 (Sigma-Aldrich, T8787) was used at 1% in phosphate-buffered saline (PBS) after pH adjustment. The Triton X-100-treated cells were applied at 37°C for 1 h. All the procedures were followed as described (Michaux et al., 2006). The v-ATPase inhibitor, bafilomycin A1 (Abcam, ab120497), in 200 nM final concentration or 0.1% DMSO was added into the growth medium, respectively, washing the cells with growth medium twice after 1 h and then fixing them with 4% PFA immediately.

### RNA Interference

*HPS6* expression was suppressed with a siRNA (5′-GCU​GGA​GAG​GAA​GGU​CCU​ATT-3′). *ATP6V0D1* expression was suppressed with a siRNA (5′-GCA CUG AUU AUG GUA ACU UTT-3′). The results were compared with a non-targeting NC siRNA (5′-UUC​UCC​GAA​CGU​GUC​ACG​UTT-3′). A total of 120 nM siRNAs were introduced into HUVECs using Lipofectamine RNAiMAX transfection reagent (Thermo Fisher Scientific, 2103411) according to the protocol of the manufacturer. After transfection (72 h), the cells were treated as needed.

### Western Blotting

Cells were lysed with cell lysis buffer for Western and IP [Beyotime, P0013, 20 mM Tris (pH 7.5), 150 mM NaCl, 1% Triton X-100] supplemented with protease inhibitor mixture (Solarbio Life Science, P6730) on ice for 1 h. The lysates were separated in sodium dodecyl sulfate (SDS)-PAGE gels [30% acrylamide, 1.5 M Tris-HCl (pH 8.8), 1.0 M Tris-HCl (pH 6.8), 10% SDS, 10% ammonium persulfate, and TEMED] and then transferred to a polyvinylidene difluoride membrane (Millipore, IPVH00010), which was blocked with 5% non-fat milk in Tris-buffered saline and 0.2% Tween-20 (TBST) for 1 h at room temperature, followed by overnight incubation at 4°C with primary antibodies. After three washes with TBST, the membrane was incubated with appropriate horseradish peroxidase-conjugated secondary antibodies for 1 h at room temperature. The antigen was detected with Mini Chemiluminescent Imaging and Analysis System (MiniChemi) according to the instructions of the manufacturer. The procedures were repeated with the same samples more than twice to react with each primary antibody.

### Immunochemistry

Cultured cells were washed once with PBS and fixed in 4% PFA. The cells were permeabilized with 0.4% Triton X-100 in PBS for 20 min and then blocked with 1% bovine serum albumin (BSA) in PBS for 2 h at room temperature. After blocking, the cells were incubated overnight at 4°C with primary antibodies against vWF (diluted 1:2,000 in 3% BSA) and Myc tag (diluted 1:1,000 in 3% BSA). Alexa red- or green-labeled secondary antibodies (1:2,000) were used for signal detection. To stain the nucleus, the cells were incubated with DAPI (ZSGB-BIO, ZLI-9557) before mounting. The labeled cells were observed with a confocal laser scanning microscope (ZEISS, LSM880) equipped with 405-, 488-, and 561-nm excitation laser. Images were taken with ×100 oil objective lens (Zeiss) using ZEN-Black (Zeiss).

### vWF Multimer Analysis

Samples were gained from the supernatant after PMA stimulation as described above. In this experiment, the same amount of cells was seeded into each well of the 24-well plate, and the same volume of medium (200 μl) was used during the PMA stimulation. After treating with 80 nM PMA in each well for 1 h, the supernatant samples (150 μl) were loaded in 50 mmol/L Tris, pH 8.0, 1% SDS, 5% glycerol, and 0.002% bromophenol blue. The vWF multimer analysis was as previously described (Ma et al., 2016). Then, 1.2% agarose gels were prepared by dissolving Seakem high-gelling-temperature agarose (Lonza, 50,041) in 0.375 mol/L Tris (pH 8.8), with SDS added to a final concentration of 0.1%. The gels were run at 30 V for 16 h (Tanon, EPS600) before transferring to a nitrocellulose membrane labeled with rabbit anti-vWF antibody (1:10,000), followed by horseradish peroxidase-conjugated anti-rabbit secondary antibody (1:5,000), and developed by chemiluminescence (Meilunbio, MA0186). The multimers of each lane on the same gel were arranged according to their molecular weight. The multimer gels were analyzed using the NIH ImageJ software. The quantification of supernatant vWF multimers was carried out based on the normalization of the β-actin protein level of the cells in each well.

### Plasmid Transfection

The coding sequence of *ATP6V0D1* was amplified by PCR from human cDNA and cloned into the pCDNA3.1 Myc HisB vector, generating *ATP6V0D1* constructs tagged with Myc at the C-terminus. The Flag-HPS6 was obtained from the lab of Jiajia Liu (Li et al., 2014). The plasmids were transfected into 293 T cells with Lipofectamine 2000 (Invitrogen, 11,668) according to the protocol of the manufacturer. The plasmids were transfected into HUVECs with Lipofectamine 3000 (Invitrogen, L3000-015) according to the protocol of the manufacturer.

### Co-immunoprecipitation Assay

Co-IP assays were performed as described by Zhang *et al*. (2014). The transfected 293 T cells were harvested and lysed with lysis buffer (1 M Tris-HCl, pH 7.4, 5 M NaCl, 0.5 M EDTA, 1% Triton X-100, and protease inhibitors). The cell lysates were centrifuged at 12,000 rpm for 5 min, and the supernatant was collected and incubated overnight with anti-FLAG M2 affinity antibody (Sigma-Aldrich, A2220) at 4°C and washed 4 times with ice-cold lysis buffer. The samples were eluted with protein loading buffer (Solarbio, P1040) and subjected to SDS-PAGE and Western blotting with anti-Myc antibody (1:1,000) or anti-Flag tag antibody (1:1,000).

### OptiPrep Continuous Density Gradient Centrifugation

The fractionation assay was performed using the OptiPrep gradient method as described by Zhang *et al*. (2014). The HUVECs were washed once with PBS and transferred to a 15-ml centrifuge tube after digestion with trypsin. The supernatant was discarded after centrifugation at 1,000 rpm for 3 min. The sample was resuspended and transferred to a 1.5-ml Eppendorf tube. The supernatant was discarded again after centrifugation. The sample was immediately homogenized with 250 μl HB lysis buffer (250 mM sucrose, 20 mM Tris-HCl, pH 7.4, and 1 mM EDTA) and ground. The sample was placed onto the top of a 12.4-ml continuous 5–50% OptiPrep (Axis-Shield, 1,114,542) gradient in HB buffer. The gradient was centrifuged at 30,000 g for 16 h in a Beckman SW41 rotor at 4°C. Thirteen fractions (900 μl each) were collected from the top. Equal aliquots from each fraction were analyzed for Western blotting.

### Statistical Analysis

The NIH ImageJ software was used for the quantitative analysis of Western blotting, determining the number of WPBs in a single cell, and the measurement of Feret’s diameter. All results were independently repeated at least three times. All the histograms were plotted using GraphPad Prism software. Student’s *t*-test was used as the statistical method, and mean ± standard error (SEM) was used to represent the data. *p < 0.05 indicated that there were significant differences in the data. **p < 0.01 and ***p < 0.001 indicated that there was a very significant difference in the data, while NS indicates that there is no significant difference in the data.

## Data Availability

The raw data supporting the conclusion of this article will be made available by the authors without undue reservation.
